# Efficiency of Semen Cryopreservation in Stallions

**DOI:** 10.3390/ani10061033

**Published:** 2020-06-13

**Authors:** Jörg Aurich, Juliane Kuhl, Alexander Tichy, Christine Aurich

**Affiliations:** 1 Obstetrics, Gynaecology and Andrology, Department for Small Animals and Horses, Vetmeduni Vienna, 1210 Vienna, Austria; joerg.aurich@vetmeduni.ac.at; 2 Artificial Insemination and Embryo Transfer, Department for Small Animals and Horses, Vetmeduni Vienna, 1210 Vienna, Austria; juliane.kuhl@vetmeduni.ac.at; 3 Bioinformatics and Biostatistics, Department for Biomedical Science, Vetmeduni Vienna, 1210 Vienna, Austria; alexander.tichy@vetmeduni.ac.at

**Keywords:** breed, cryopreservation, horse, semen

## Abstract

**Simple Summary:**

The quality of stallion semen after freezing and thawing differs among stallions, but it remains to be determined whether such differences are also affected by horse breed. In this study, semen quality was analysed retrospectively in 1012 frozen–thawed ejaculates from 134 stallions of 5 breeds. The percentage of ejaculates acceptable for artificial insemination (AI) and the number of insemination doses per ejaculate was then calculated. Sperm motility before freezing was the most important explanatory variable for the percentage of ejaculates with a post-thaw quality acceptable for AI. Of the other variables studied, stallion age was the most important parameter and a decrease in the percentage of acceptable ejaculates was evident in stallions older than 9 years. There were also more acceptable frozen–thawed ejaculates in Arab stallions compared to Warmbloods, Quarter Horses and Icelandic horses. The analysis thus demonstrates differences in the percentage of acceptable cryopreserved ejaculates among breeds. Total sperm count was the most important variable determining the number of semen doses obtained per acceptable frozen–thawed ejaculate.

**Abstract:**

Differences in the cryotolerance of spermatozoa exist among stallions, but it remains to be determined to what extent such differences are affected by breed. In this study, post-thaw semen quality in stallions presented for semen cryopreservation was analysed retrospectively (1012 ejaculates from 134 stallions of 5 breeds). The percentage of frozen–thawed ejaculates acceptable for artificial insemination (AI) and the number of insemination doses per cryopreserved ejaculate was calculated. Logistic regression analysis revealed sperm motility in raw semen as the most important explanatory variable for the percentage of cryopreserved ejaculates with a post-thaw quality acceptable for AI. Of the other variables included into the model, stallion age was the most important parameter with more acceptable ejaculates in younger than in older stallions. Logistic regression also showed more acceptable frozen–thawed ejaculates in Arab stallions versus Warmbloods, Quarter Horses and Icelandic horses. The analysis thus demonstrates differences in the percentage of acceptable cryopreserved ejaculates among horse breeds. Season was a less relevant explanatory variable for percentage of acceptable cryopreserved ejaculates. Logistic regression revealed total sperm count as the most important variable determining the number of cryopreserved semen doses obtained per acceptable ejaculate. In conclusion, logistic regression analysis revealed stallion age and breed as explanatory variables for the percentage of cryopreserved ejaculates acceptable for AI.

## 1. Introduction

In equine artificial insemination (AI), cryopreserved semen is used far less frequently than fresh or cooled-stored semen [1,2]. This is at least in part due to differences in the fertilizing capacity of frozen–thawed semen among stallions [3,4,5,6]. Furthermore, cryopreservation of semen is associated with substantial costs. The number of insemination doses obtained per ejaculate thus contributes to the economic efficiency of commercial AI with cryopreserved semen. The efficiency of semen cryopreservation in commercial equine AI programs has not, to the best of our knowledge, been investigated so far. Differences in the number of cryopreserved insemination doses per ejaculate exist among individual stallions, but it remains to be determined whether such differences are also affected by horse breed. Although semen has been frozen from a large number of breeds, ranging from draught horses [7] to Shetland ponies [8,9], differences among horse breeds in post-thaw motility and sperm DNA fragmentation have only recently been investigated in one study [10]. In that study, except for a slightly lower sperm DNA fragmentation in semen frozen from Arab compared to Thoroughbred stallions, post-thaw semen quality did not differ among Arab, Thoroughbred and Warmblood stallions. To the best of our knowledge, the efficiency of semen cryopreservation, i.e., the percentage of frozen–thawed ejaculates classified as acceptable for commercial AI and the number of insemination doses obtained per ejaculate, have not been analysed when using the same cryopreservation protocol in different breeds. However, this is of increasing interest as AI with cryopreserved semen is becoming an alternative in more and more horse breed registries.

Under commercial conditions, the number of cryopreserved semen doses per ejaculate is of great interest, provided post-thaw semen quality is of acceptable quality. Preliminary data from our group indicate that the production of cryopreserved semen in horses might be most efficient during the summer months [11,12]. Seminal plasma affects the quality of cryopreserved stallion semen [5]. Seasonal variations in seminal plasma composition may therefore contribute to changes in the quality of processed semen. Additionally, seasonal changes in the composition of the equine sperm plasma membrane have to be considered [13].

Stallions reach their full spermatogenetic capacity not before an age of six years [14] and, furthermore, post-thaw sperm motility was lower in stallions up to six years of age than in older stallions [10]. Additionally, in cattle, total sperm motility, membrane integrity and concentration of motile spermatozoa determined after freezing-thawing were higher in 4-year-old than in yearling bulls [15]. In sheep, frozen–thawed semen from mature rams had less hyperactivated and peroxidised spermatozoa and higher percentages of viable non-capacitated spermatozoa, spermatozoa with an intact plasma membrane, functional mitochondria and condensed chromatin than frozen–thawed semen from young rams. Consequently, lambing rate was higher in ewes inseminated with frozen–thawed semen from mature versus young rams [16].

In the present study, we have retrospectively analysed post-thaw semen quality in stallions admitted to the Centre for Artificial Insemination and Embryo Transfer at Vetmeduni Vienna for semen cryopreservation. The percentage of frozen–thawed ejaculates meeting recommendations for commercial use in equine AI and the number of insemination doses obtained per cryopreserved ejaculate was then calculated. We hypothesized that the percentage of acceptable frozen ejaculates and thus the economic efficiency of equine semen cryopreservation depends on breed of the stallion, increases with age of the stallion until sexual maturity and differs among ejaculates frozen at different times of the year.

Classification and regression trees (CART) analysis was used for determination of a relationship between the percentage of ejaculates acceptable for AI and age and breed of stallion as well as season. Classification and regression trees are a nonparametric method used to explain the variation in a response variable with one or more explanatory variables. The analysis separates the response variable into two groups based on one of the explanatory variables. Further splits are obtained in the same way within each of the first two groups [17]. Furthermore, the individual stallion and pre-freeze semen motility were analysed as explanatory variables. 

## 2. Materials and Methods

### 2.1. Animals

Semen characteristics in raw, diluted and frozen–thawed semen from ejaculates (n = 1221) collected from 164 stallions referred to the Centre for Artificial Insemination and Embryo Transfer of Vetmeduni Vienna between 2004 and 2016 were analysed retrospectively. All stallions underwent a breeding soundness examination before and were only included into the cryopreservation programme when raw semen characteristics at this examination met minimal requirements (>70% motile spermatozoa, sperm concentration >100 × 10^6^/mL, total sperm count >5 × 10^9^). Semen was cryopreserved following European Union regulations (Directive 65/92 EEC) and stallions were therefore tested serologically negative for equine infectious anaemia and negative for contagious equine metritis based on PCR performed on two sets of penile swabs collected at a 7 day-interval. Stallions were either serologically negative for equine viral arteritis or were tested as not shedding equine arteritis virus in their semen. Decisions on the number of ejaculates and/or insemination doses to be frozen were made by the owner of the individual stallion. Only breeds with more than seven stallions were included into the analysis, leaving 1012 ejaculates from 134 stallions and five breeds. The breeds, number of stallions and number of ejaculates included in the study are summarised in Table 1 and Table 2. 

Ejaculates were grouped according to the stallion’s age at time of semen freezing (2–4 years: n = 179, 5–9 years: n = 331, 10–14 years: n = 240; >14 years: n = 262), season of semen collection (Spring: March–May, n = 325; Summer: June–August, n = 63; Autumn: September–November, n = 240; Winter: December–February, n = 384).

Stallions were housed in individual boxes with access to an outdoor paddock for two to three hours per day. The stallions were fed hay and concentrates three times daily and water was provided ad libitum. Because semen collections were performed as part of regular commercial AI programmes and were neither related to the study nor to other experimental purposes, no animal experimentation approval by the competent authority was required.

### 2.2. Semen Collection and Cryopreservation

Semen collection and freezing always followed the same protocol. Semen was collected with an artificial vagina (Hannover model; Minitube, Tiefenbach, Germany) three times a week (Monday, Wednesday and Friday) on a dummy in the presence of a teaser mare displaying oestrous behaviour. Immediately after collection, the gel fraction of the ejaculate was removed. Semen was filtered through sterile gauze, and ejaculate volume and colour were determined. Sperm concentration was measured by SpermaCue (until 2009; Minitube) or NucleoCounter (from 2010 onwards; ChemoMetec, Allerød, Denmark). Semen was analysed as described previously [18]. In brief, the percentage of motile (total motility), progressively motile spermatozoa, and membrane-intact spermatozoa was evaluated with a computer-assisted sperm analyser (CASA; SpermVision, Minitube) as described [19,20]. Spermatozoa with average orientation change <8 μm were considered immotile. Spermatozoa with curvilinear velocity ≥10 μm/s, distance straight line ≥6 μm and radius ≥15 μm were considered progressively motile. For assessment of sperm membrane integrity, 100 μL of semen was mixed with 2 μL of SYBR-14/propidium iodide and incubated for 10 min at room temperature in darkness. One droplet was placed onto a glass slide, covered with a glass coverslip, and evaluated by fluorescence microscopy at magnification × 400 (Olympus AX70; Olympus, Vienna, Austria; U-MWB filter block, BP420–480 excitation filter, BA515 suppressor filter, dichromatic mirror: DM500). Heads of membrane-intact (viable) spermatozoa were stained with a bright green colour; spermatozoa with damaged membranes were stained red. Spermatozoa were recognised by the video camera of SpermVision according to their colour. At least 15 fields were evaluated, and the average value was calculated by the CASA system.

Semen cryopreservation followed an established protocol [9]. The semen was diluted 1:1 with EquiPlus extender (Minitube). The diluted semen was centrifuged at 700 g for 12 min, the supernatant was aspirated, and the pellet was resuspended at a ratio of 1:1 with Ghent freezing extender (Minitube). Final concentration of glycerol in extended semen was 2.5%. The semen was filled into 0.5-mL straws and straws were sealed automatically at room temperature (MPP Uno, Minitube). Straws were placed on a rack into the freezing chamber of a computer-controlled rate freezer at 20 °C (IceCube 14 M; Sylab, Purkersdorf, Austria). Semen was first cooled to 5 °C at a cooling rate of 0.3 °C/min, subsequently within 3 min to −25 °C (10 °C/min) and finally to −140 °C at a cooling rate of 25 °C/min. Straws were removed from the freezing chamber and plunged directly into liquid nitrogen where they were stored for at least 24 h before thawing.

From each ejaculate, at least one straw was used for determination of post-thaw semen characteristics. The straw was thawed in a water bath at 37 °C for 15 s. The thawed semen was transferred to an Eppendorff tube and left at room temperature for at least 10 min before further analysis. Post-thaw quality of semen was evaluated for total and progressive motility as well as sperm membrane integrity as described above. In accordance with WBFSH recommendations [21], semen was classified acceptable for insemination when progressive motility after thawing was ≥35%. One insemination dose consisted of at least 250 × 10^6^ progressively motile spermatozoa after thawing.

### 2.3. Statistical Analysis

Statistical analysis was performed with the SPSS statistics package (version 24.0, IBM-SPSS, Armonk, NY, USA). After confirmation of normal distribution and homogeneity of variances, pre-freeze semen parameters were compared among horse breeds by ANOVA. The impact of age and breed of the stallion and season on the percentage of frozen–thawed ejaculates acceptable and non-acceptable for AI was assessed using logistic regression analyses. In addition, a CART (classification and regression tree) analysis was performed. Several models were calculated adding stallion and pre-freeze semen motility to the models. The aim of these analyses was to classify semen as acceptable or non-acceptable for AI by a specific combination of these variables. The overall performance is expressed as the accuracy (percentage of correct classifications). As a result, variables used in the tree are ranked according to their importance for the classification process. The importance of each variable is expressed relative to the most important variable, which is normalized to 100%. The CART analysis is a descriptive way to find a classification algorithm which is easy to understand and helps to generate a deeper insight in the structure of the relationship between the variables used in the model. Data are expressed as means ± standard deviation (SD) and *p*-values < 0.05 were considered significant.

## 3. Results

Ejaculate volume, sperm concentration and total sperm count in raw semen differed among breeds (ejaculate volume and sperm concentration *p* < 0.05, total sperm count *p* < 0.001; Table 2). Ejaculate volume was highest in Lipizzaner and lowest in Quarter Horse stallions, whereas sperm concentrations was lowest in Quarter Horses and highest in Arabs. Total sperm count was highest in Warmblood and lowest in Quarter Horse stallions. Neither total and progressive motility nor membrane integrity before freezing differed significantly among breeds (Table 2). In frozen–thawed ejaculates, post-thaw motility did not differ significantly among breeds. For post-thaw membrane integrity breed differences existed (*p* = 0.05) with lowest values in Icelandic stallions and highest in Warmblood stallions (Table 2).

Out of the 134 stallions in this study, 59 produced only cryopreserved ejaculates classified as acceptable for AI (progressive motility ≥35%), 26 stallions had only non-acceptable cryopreserved ejaculates and in 49 stallions both cryopreserved ejaculates acceptable and non-acceptable for AI were produced.

The percentage of frozen–thawed ejaculates classified as acceptable for AI was below the average of all stallions in Quarter Horses and Icelandic horses and above average in Arab stallions. More insemination doses were obtained from Warmblood, Arab and Lipizzaner stallions than from Quarter Horse and Icelandic stallions. When stallions were grouped by age, the percentage of frozen–thawed ejaculates acceptable for AI was higher than the overall average in stallions aged 5 to 9 years and lower than average in stallions aged 10 to 14 years. The number of insemination doses per ejaculate was lowest in stallions aged 10 to 14 years (Table 3). The percentage of acceptable ejaculates after cryopreservation (defined as post-thaw progressive motility ≥35%) was affected by pre-freeze total and progressive semen motility but also by age and breed of the stallion, while season within the year and total sperm count of the ejaculate had least influence. Normalized independent variable importance obtained by logistic regression is summarised in Table 4.

**Figure 1:**
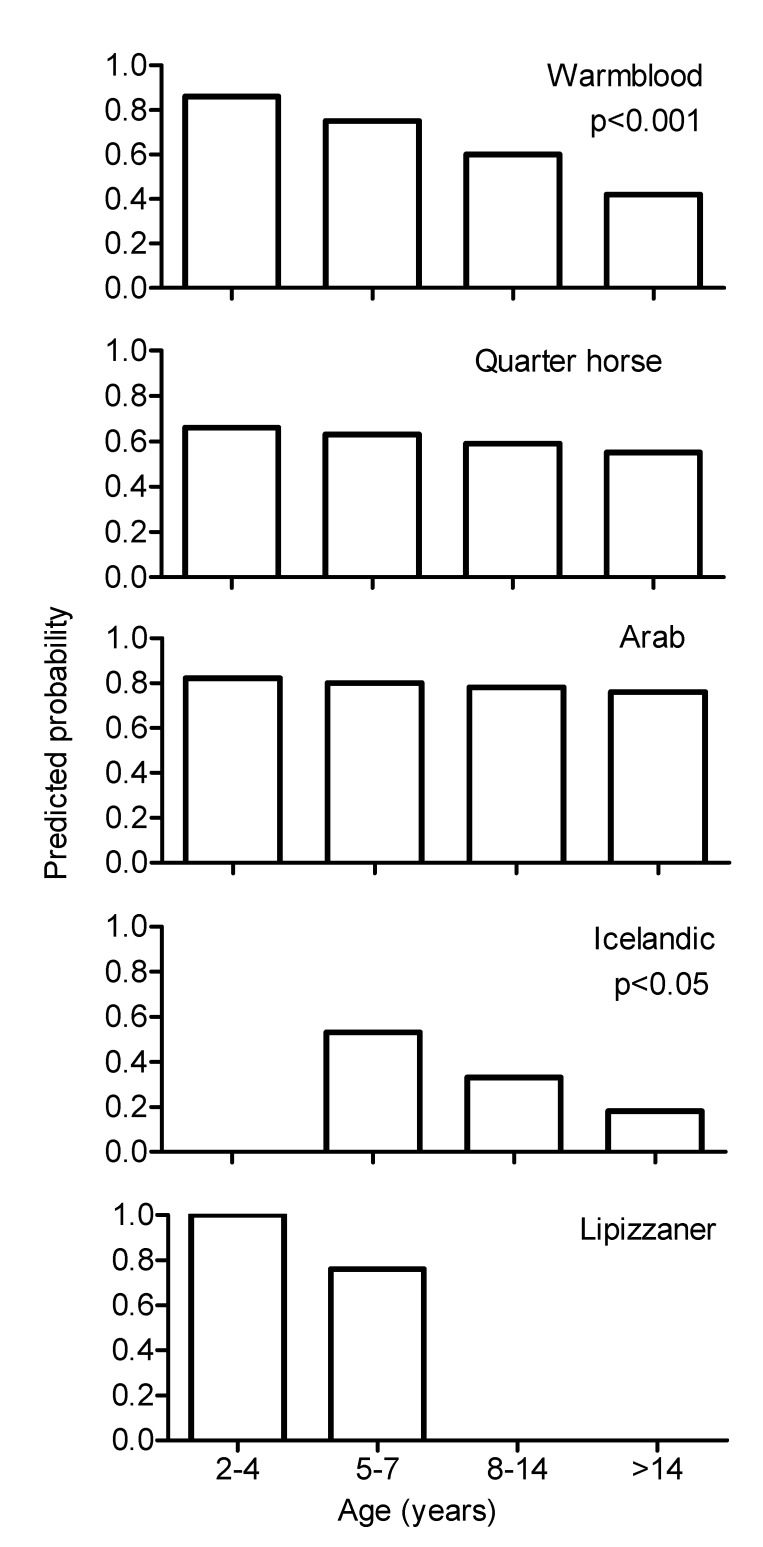
Aggregated probability for acceptable cryopreserved ejaculates (progressive motility after freezing-thawing ≥35%) determined by logistic regression analysis for breed and age of stallion, decrease with age in Warmblood (*p* < 0.001) and Icelandic stallions (*p* < 0.05).

Logistic regression revealed total sperm count as the most important independent variable determining the number of cryopreserved semen doses for artificial insemination obtained per acceptable ejaculate. Further independent variables were, in declining order, total motility, progressive motility and membrane integrity in fresh semen and breed of the stallion, while the relative importance of season and age of stallion was only approximately 1% (Table 4 and Figure 1).

**Figure 2:**
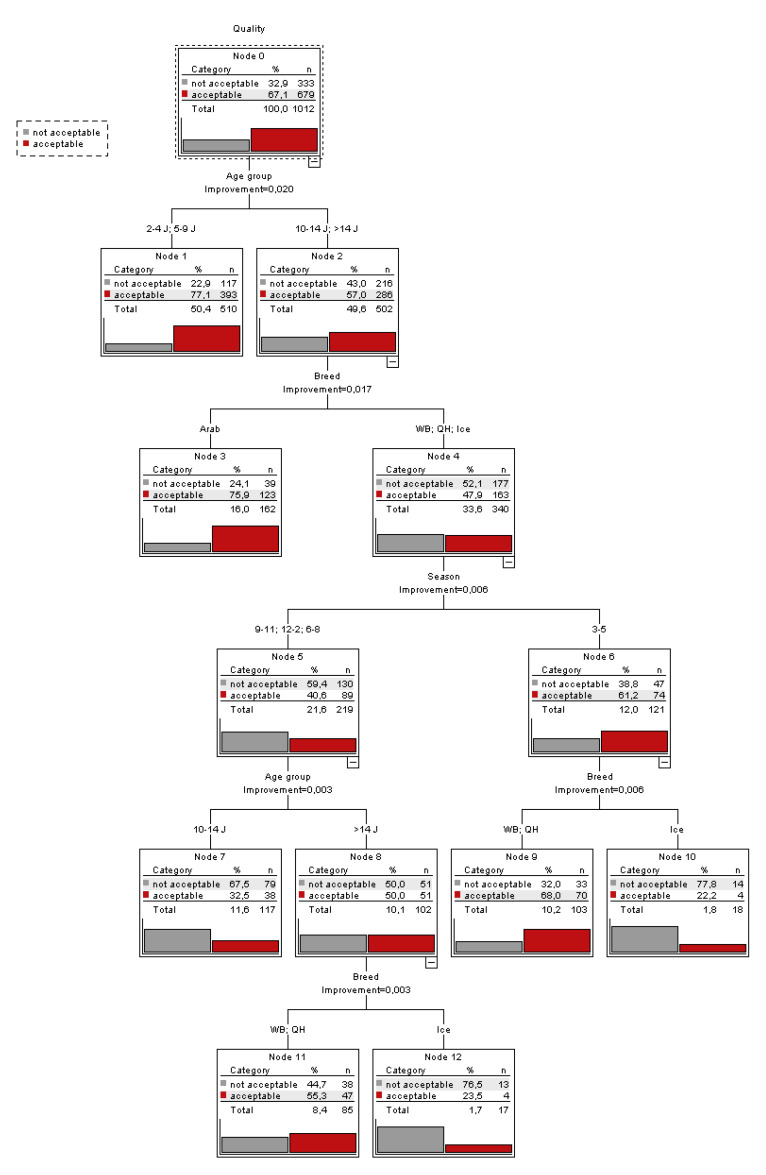
Classification tree prediction percentage of acceptable and non-acceptable ejaculates after cryopreservation determined by age group and breed of the stallion. No seasonal effect was shown.

Classification tree analysis for age group and breed of the stallion and season of semen freezing revealed that age group was the most important parameter determining the percentage of frozen–thawed ejaculates acceptable for AI with more acceptable ejaculates in younger than in older stallions (Figure 2). The second node of the classification trees showed more acceptable frozen–thawed ejaculates in Arab stallions versus Warmbloods, Quarter Horses and Icelandic horses and subsequent splits showed further effects of season, age and breed (Figure 2). Ejaculates frozen in summer were classified more often as acceptable for AI than ejaculates frozen at any other season.

## 4. Discussion

With the costs of collecting and freezing one ejaculate as a largely constant value, the economic efficiency of semen cryopreservation is in part determined by the percentage of frozen–thawed ejaculates deemed acceptable for use in commercial AI programmes and by the number of semen doses obtained per acceptable ejaculate. In agreement with European studbook recommendations [21], in our study frozen–thawed semen was classified acceptable for insemination when progressive motility was ≥35%. This is also in good agreement with standards defined in the United States [6]. Even if a stallion provides semen characteristics that allow considering his semen acceptable for cryopreservation, not all ejaculates collected during a cryopreservation trial will meet minimal requirements after freezing-thawing [6].

Not surprisingly, logistic regression analysis revealed sperm motility in raw semen as the most important explanatory variables for the percentage of ejaculates classified as acceptable for AI after freezing and thawing in the present study. Stallions with good semen quality directly after collection are therefore on average more likely to yield acceptable semen after cryopreservation than stallions with an already reduced quality of raw semen. Warmblood stallions have been suggested suitable for semen cryopreservation when raw semen ejaculates have a sperm concentration of at least 200 million per ml, at least 50% progressive motility and at least 70% morphologically normal spermatozoa [22]. Nevertheless, good sperm motility in raw semen not always correlates with similar post-thaw semen quality, and our data do not contradict the finding of individual “bad freezer” stallions despite acceptable quality of raw semen [22]. In agreement with previous reports [6] approximately 20% of the stallions in our study were consistent “bad freezers”.

The next important explanatory variable for the percentage of cryopreserved ejaculates classified as acceptable for AI was stallion age. A decrease in raw semen quality with increasing age of the stallion has been documented previously [14], and our data extend these findings to cryopreserved semen. Thus, the risk that ejaculates do not fulfil quality standards for cryopreservation increases with age of the stallion. A decrease in the percentage of acceptable cryopreserved ejaculates became already evident in stallions older than 9 years. In stallions with high breeding values obtained through success in equestrian competitions, semen cryopreservation should therefore preferably be done at a younger age and not after the stallion has been retired from a career in equestrian sports. A marked variation in the quality of raw semen with stallion age has been demonstrated previously [14,23], and the percentage of acceptable cryopreserved ejaculates declined in stallions over 11 years of age, which is in agreement with the results of our study. There was, however, no difference in post-thaw semen characteristics and the number of AI doses per ejaculate between stallions aged 2 to 4 years and those aged 5 to 9 years. Production of cryopreserved semen in stallions is therefore already possible also with regard to economic considerations in stallions that have not yet reached complete sexual maturity, which does not occur before an age of six years [14]. This result is thus not in agreement with findings from ruminants where characteristics of frozen–thawed semen improved until males reached maturity [15,16]. In partial contrast to our study, a lower post-thaw progressive motility in semen from 2- to 6-year old compared to older stallions has been reported recently [10]. Because overall post-thaw progressive motility in that study was approximately 10% lower than in our stallions, results, however, might not be directly comparable.

Furthermore, our study demonstrates differences in the percentage of acceptable cryopreserved ejaculates and, thus, the economic efficiency of semen cryopreservation among horse breeds. Differences among individual stallions in cryotolerance of their semen are well known [3,4,5,6,10] and sires have been divided into good and bad freezers based on a threshold of 35% progressively motile spermatozoa after freezing [5]. Breed effects on the percentage of cryopreserved ejaculates acceptable for use in commercial AI programmes to the best of our knowledge have not been compared in one study before. This percentage was above the expected average in Arab stallions, below the average in Icelandic and Quarter Horse stallions and close to the average in Warmblood and Lipizzaner stallions. While the number of Icelandic horses and Lipizzaners in our study may be considered relatively small, breed differences between Arab, Quarter Horse and Warmblood stallions are based on a substantial number of animals and ejaculates. Breed differences in parameters such as volume, sperm concentration and total sperm count in raw semen have been demonstrated repeatedly [6,24], and were also evident for the pre-freeze ejaculates of the present study. Conflicting evidence exists regarding breed differences in raw semen sperm motility, and while these were absent in the stallions of our study, minor differences among breeds have been reported by others [6]. This slight difference may be due to the fact that stallions in our study represented a preselected population of AI stallions and more pronounced differences have to be expected in unselected stallion populations.

Our study did not aim to investigate the mechanisms behind age and breed differences in post-thaw semen motility. Sperm membrane lipid composition affects the resistance of spermatozoa to cryopreservation [11] and we could recently demonstrate an increase in sperm membrane polyunsaturated fatty acid (PUFA) content from the non-breeding into the breeding season [13]. In cattle, cryopreserved semen samples defined as good quality differed from semen with lower quality in lipid concentration and fatty acid composition in both seminal plasma and the cell compartment [25]. An active lipid metabolism has recently been demonstrated in the stallion testis and epididymis and most likely affects the lipid profile of the equine sperm plasma membrane [26]. The individual composition of seminal plasma also affects the suitability of stallions for semen cryopreservation [5] and may change to some extent with age and differ among breeds. With regard to breed differences, a genetic basis appears feasible and might manifest itself in differences in lipid metabolism, the sperm membrane lipid pattern and the composition of seminal plasma. In the present study, differences in feeding or management of stallions among breeds at least during the cryopreservation trials can be excluded. At our semen collection centre, stallions were kept under identical conditions and fed the same diet. Stallions selected for semen cryopreservation are always considered valuable animals by their owners, and it can be assumed that such stallions are well and adequately fed and housed also in their home stud. To the best of our knowledge, their diet was not supplemented with any specific components before they were referred to the AI centre.

Season of semen processing was a less relevant explanatory variable for percentage of acceptable cryopreserved ejaculates than age and breed of the stallion. Seasonal effects were, however, evident in the classification tree analysis for Quarter Horse and Icelandic stallions, i.e., two breeds with apparently lower cryotolerance of spermatozoa. In these breeds, summer was the best season for semen cryopreservation in the present investigation. It has recently been demonstrated that seasonal changes in sperm fatty acid composition might in part explain seasonal differences in the resistance of equine spermatozoa to cryopreservation and cooled-storage [11,13] but such effects may also depend on breed. In most stallions, successful semen cryopreservation outside the breeding season is possible.

In agreement with Warmblood breed registry recommendations [21], one insemination dose consisted of at least 250 × 10^6^ progressively motile spermatozoa after thawing. As predefined by this fixed number of spermatozoa per insemination dose, total sperm count per acceptable cryopreserved ejaculate was the most important explanatory variable for the number of insemination doses obtained per ejaculate. Motility and membrane integrity in fresh semen and breed of stallion also affected the number of insemination doses per ejaculate, while age of the stallion and season were least relevant. A certain breed effect primarily reflects differences in total sperm count among horse breeds and, in particular, a lower sperm count in Quarter Horse compared to Warmblood stallion ejaculates.

## 5. Conclusions

Logistic regression analysis revealed stallion age and breed as explanatory variables for the percentage of cryopreserved ejaculates acceptable for AI. The risk that ejaculates do not fulfil quality standards for cryopreservation thus increases with age of the stallion. Differences in the percentage of acceptable cryopreserved ejaculates among horse breeds suggest a genetic basis for cryotolerance of equine semen. Total sperm count per ejaculate was the most important explanatory variable for the number of cryopreserved insemination doses obtained per ejaculate.

## Figures and Tables

**Table 1:** Horse breeds included into the study with number of stallions and number of cryopreserved ejaculates per stallion.

Breed	Number of Stallions (n)	Number of Ejaculates (n)	Number of Ejaculates per Stallion (mean ± SD)	Minimal Number of Ejaculates	Maximal Number of Ejaculates
Warmblood	57	330	5.8 ± 4.8	1	19
Quarter Horse	34	319	9.4 ± 9.5	1	41
Arab	26	243	9.3 ± 7.9	1	36
Icelandic	9	74	8.2 ± 5.7	4	21
Lipizzaner	8	45	5.8 ± 5.2	1	17

**Table 2:** Semen characteristics (*n* = 134 stallions and 1012 ejaculates) for different breeds before freezing and after thawing (mean ± SD).

				Pre-Freeze			Post-Thaw		
Breed	Volume (mL)	Concentration (mio/mL)	Total Sperm Count (× 10^9^)	Total Motility (%)	Progressive Motility (%)	Membrane Integrity (%)	Total Motility (%)	Progressive Motility (%)	Membrane Integrity (%)
Warmblood	41 ± 27	322 ± 182	10.3 ± 4.6	83 ± 12	61 ± 18	85 ± 10	60 ± 15	43 ± 16	53 ± 12
Quarter Horse	29 ± 21	240 ± 117	5.8 ± 2.9	83 ± 9	61 ± 15	84 ± 8	59 ± 16	41 ± 17	51 ± 13
Arab	33 ± 20	337 ± 238	8.2 ± 3.5	86 ± 9	70 ± 16	85 ± 11	61 ± 15	47 ± 18	50 ± 15
Icelandic	35 ± 18	242 ± 97	8.0 ± 4.7	79 ± 12	56 ± 17	82 ± 9	47 ± 16	29 ± 14	41 ± 12
Lipizzaner	51 ± 24	227 ± 153	10.0 ± 4.8	88 ± 7	68 ± 15	87 ± 5	59 ± 16	42 ± 13	45 ± 12

**Table 3:** Percentage of ejaculates acceptable for artificial insemination (AI) after cryopreservation (post-thaw progressive motility ≥35%) and number of insemination doses (250 × 10^6^ progressively motile spermatozoa/dose; mean ± SD) in stallions of different breed and age and ejaculates frozen at different times of the year.

Breed	Warmblood	Quarter Horse		Arab		Icelandic		Lipizzaner
Acceptable for AI (%)	70.3	60.5		77.8		37.8		80.4
AI doses (n)	10.5 ± 7.3	5.8 ± 4.3		9.3 ± 6.7		4.8 ± 3.9		9.6 ± 6.7
Age group	2–4 years		5–9 years		10–14 years		≥15 years	
Acceptable for AI (%)	75.4		78.0		50.0		63.7	
AI doses (n)	9.2 ± 6.0		9.3 ± 6.8		6.4 ± 4.9		8.0 ± 7.2	
Season	Spring		Summer		Autumn		Winter	
Acceptable for AI (%)	71.2		81.0		73.3		57.0	
AI doses (n)	7.9 ± 5.7		10.5 ± 7.7		9.2 ± 6.5		7.6 ± 6.7	

**Table 4:** Independent variable importance for the dependent variable percentage of acceptable ejaculates after cryopreservation and number of insemination doses per acceptable ejaculate.

Percentage of Acceptable Ejaculates	Importance	Normalized Importance (%)
Total motility in fresh semen	0.191	100.0
Progressive motility in fresh semen	0.190	99.9
Membrane integrity in fresh semen	0.120	62.8
Age group of stallion	0.036	19.0
Breed of stallion	0.023	12.0
Season	0.013	7.0
Total sperm count/ejaculate	0.010	5.3
Insemination doses per acceptable ejaculate		
Total sperm count/ejaculate	24.804	100.0
Total motility in fresh semen	11.242	45.3
Progressive motility in fresh semen	8.675	35.0
Membrane integrity in fresh semen	7.122	28.7
Breed of stallion	4.717	19.0
Season	0.285	1.1
Age group of stallion	0.268	1.1
